# RETRACTION: Long Non‐Coding RNA CRNDE Sponges miR‐384 to Promote Proliferation and Metastasis of Pancreatic Cancer Cells through Upregulating IRS1

**DOI:** 10.1111/cpr.13690

**Published:** 2024-06-10

## Abstract

G. Wang, J. Pan, L. Zhang, Y. Wei, C. Wang, “Long Non‐Coding RNA CRNDE Sponges miR‐384 to Promote Proliferation and Metastasis of Pancreatic Cancer Cells through Upregulating IRS1,” *Cell Proliferation* 50, no. 6 (2017): e12389, https://doi.org/10.1111/cpr.12389

The above article, published online on 21 September 2017 in Wiley Online Library (wileyonlinelibrary.com), has been retracted by agreement between the authors, the Deputy Editor‐in‐Chief, Yunfeng Lin, and John Wiley and Sons Ltd. The retraction has been agreed following a request by the authors to retract the article due to unreliable results and a lack of original data. Further investigation revealed multiple images previously published elsewhere in a different scientific context. Thus, the editors consider the conclusions of this manuscript substantially compromised. The corresponding author Cheng Wang agrees with this decision on behalf of all authors.

G. Wang, J. Pan, L. Zhang, Y. Wei, C. Wang, “Long Non‐Coding RNA CRNDE Sponges miR‐384 to Promote Proliferation and Metastasis of Pancreatic Cancer Cells through Upregulating IRS1,” *Cell Proliferation* 50, no. 6 (2017): e12389, https://doi.org/10.1111/cpr.12389

The above article, published online on 21 September 2017 in Wiley Online Library (wileyonlinelibrary.com), has been retracted by agreement between the authors, the Deputy Editor‐in‐Chief, Yunfeng Lin, and John Wiley and Sons Ltd. The retraction has been agreed following a request by the authors to retract the article due to unreliable results and a lack of original data. Further investigation revealed multiple images previously published elsewhere in a different scientific context. Thus, the editors consider the conclusions of this manuscript substantially compromised. The corresponding author Cheng Wang agrees with this decision on behalf of all authors.

